# Association between vitamin C in serum and trouble sleeping based on NHANES 2017–2018

**DOI:** 10.1038/s41598-024-56703-0

**Published:** 2024-04-27

**Authors:** Shihong Wang, Fengxia Lai, Le Zhao, Jiyu Zhou, Danli Kong, Haibing Yu, Yuanlin Ding

**Affiliations:** School of Public Health, Guangdong Medical University, Dongguan, China

**Keywords:** Vitamin C, Trouble sleeping, National Health and Nutrition Examination Survey, Neuroscience, Physiology, Risk factors

## Abstract

Vitamin C is an important micronutrient for human. Association between vitamin C and trouble sleeping was less studied. Therefore, the purpose of this study was to investigate the possible link between vitamin C in serum and trouble sleeping. The cross-sectional data was derived from the National Health and Nutrition Examination Survey (NHANES, 2017–2018). Trouble sleeping was measured by asking participants: “Have you ever told doctor had trouble sleeping”. Responses to this question was “yes” or “no”. vitamin C in serum was obtained by measuring the serum samples. We used multivariable binary logistic regressions to examine the possible link between vitamin C in serum and trouble sleeping, and then a subgroup analysis was performed. Moreover, the non-linear relationship between vitamin C in serum and trouble sleeping was further detected using a restricted cubic spline (RCS) model. A total of 3227 participants were included in the study. After adjusting all potential confounders, the results of multivariable logistic regression showed the significant negative association between vitamin C in serum and trouble sleeping(*OR* = 0.816; 95% *CI*:0.669 ~ 0.995). The significant inverse association was also found in female(*OR* = 0.713; 95% *CI*:0.546 ~ 0.931), age ≤ 65 years(*OR* = 0.773; 95% *CI*:0.600 ~ 0.996), and in participants with high cholesterol level(*OR* = 0.738; 95% *CI*:0.548 ~ 0.994). In addition, the RCS model demonstrated the significant non-linear relationship between vitamin C in serum and trouble sleeping (*P* value of nonlinear = 0.010). Our study demonstrates the significant negative association between vitamin C in serum and trouble sleeping.

## Introduction

Sleep is an integral part of our life^1^. According to the American Academy of Sleep Medicine and the Sleep Research Society, adults should sleep at least 7 h a night^2^. Trouble sleeping is defined as a difficulty getting to sleep or staying asleep, including insomnia, sleep deprivation, obstructive sleep apnea and other sleep problems^3,4^. With the development of society and the increase of mental stress, trouble sleeping is a common phenomenon in modern people. A previous study among US participants reported that about 14.5% of adults in America had sleeping problems^5^. Moreover, trouble sleeping is regarded as an important risk factor for many chronic diseases. Related studies have demonstrated strong associations between trouble sleeping and the occurrence of cardiovascular events, hypertension, type 2 diabetes, and depressive symptoms^3,6–8^. Currently, trouble sleeping significantly affects the life quality of a large number of people and has become a serious public health problem worldwide. Therefore, it is necessary to explore the determinants of trouble sleeping for the prevention and control of trouble sleeping.

As the factor needed for the treatment of scurvy, vitamin C (L-ascorbic acid) is an essential micronutrient for humans^9^. It is a water-soluble vitamin that is widely found in fresh fruits and vegetables^10^. Previous studies have demonstrated that vitamin C is beneficial for immune defense, antioxidant protection and prevention for cancer^11–13^. In recent years, researchers have begun to focus on the relationship between vitamin C and trouble sleeping^14^. It is well-known that vitamin C is a regulator of neurotransmitter biosynthesis. It plays an important role in the conversion of dopamine to norepinephrine, which is crucial for regulating our mood^15^. Chronic lack of vitamin C may lead to decreasing norepinephrine levels, and thus have a negative effect on our mood^16^. Meanwhile, a study suggested that prolonged periods of low mood may affect our sleep quality in a degree^17^. Therefore, whether vitamin C can help to improve trouble sleeping needs to be explored.

Rare studies have investigated the association between vitamin C in serum and trouble sleeping. We aimed at examining the association by analyzing data from the National Health and Nutrition Examination Survey (NHANES) 2017–2018.

## Materials and methods

### Study sample

**Figure 1:**
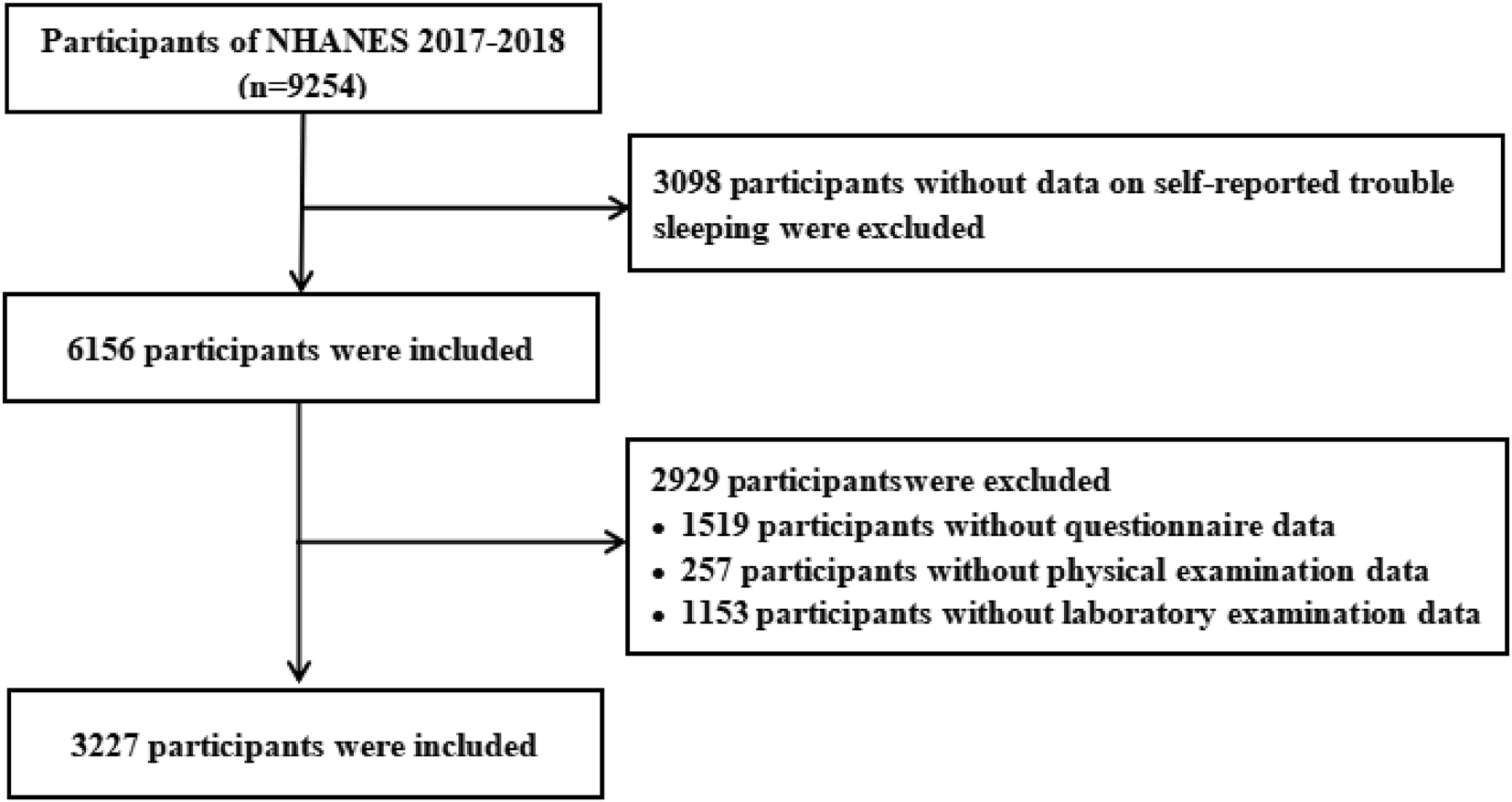
Flowchart showing the selection of study population.

The cross-sectional data is derived from the National Health and Nutrition Examination Survey (NHANES) from 2017 to 2018, involving 9254 participants. NHANES is a national and population-based study in the United States. Based on a complex multistage sampling design, NHANES obtained data on demographics, lifestyle factors and other health outcomes through interviews. The NHANES program was approved by the Ethics Review Board of the National Centre for Health Statistics (NCHS). After excluding participants with missing data, we finally included 3227 participants (Fig. 1). Complete details about NHANES can be accessed from https://www.cdc.gov/nchs/nhanes/index.htm*.*

### Self-reported trouble sleeping and vitamin C

Trouble sleeping was measured by asking participants: “Have you ever told doctor had trouble sleeping?”. Responses to this question were dichotomized as either yes or no. This question was asked in the home, by trained interviewers, using the Computer-Assisted Personal Interview (CAPI) system. Serum specimens were mixed with four parts 6% metaphosphoric acid to acidify the serum and stabilize ascorbate. All serum specimens of vitamin C were stored under appropriate frozen (− 70 °C) conditions until they were shipped to National Center for Environmental Health for testing. Vitamin C (mg/dL) in serum was measured using isocratic ultra-high performance liquid.

### Covariates

We collected 3227 participants’ baseline data and physical and laboratory examination indicators from NHANES. Baseline data included gender (male, female), age (years), race/ethnicity (Mexican American, Other Hispanic, Non-Hispanic White, Non-Hispanic Black, Other Race), diabetes (yes, no), hypertension (yes, no), high cholesterol level (yes, no), weak/failing kidneys (yes, no), little interest in doing things (not at all, several days, more than half the days, nearly every day) and feeling depressed (not at all, several days, more than half the days, nearly every day). These variables were obtained through participant self-reports. Physical examination indicators included weight (kg), height (cm), body mass index (BMI, kg/m^2^), upper leg length (cm), upper arm length (cm), arm circumference (cm) and waist circumference (cm). Leg and arm measurements were performed on the right side of the body. Measurement would be taken on the left side if a participant had an amputation or other adverse condition. Laboratory examination indicators included total cholesterol (mmol/L), high-density lipoprotein cholesterol (HDL-C, mmol/L), ferritin (ug/L), hypersensitive C-reactive protein (HS-CRP, mg/L), glycohemoglobin (%), transferrin receptor (nmol/L), alpha-carotene (ug/dL), cis-beta-carotene (ug/dL), gamma-tocopherol (ug/dL), total lycopene (ug/dL), retinol (ug/dL) and alpha-tocopherol (ug/dL). All laboratory indicators were obtained by measuring the serum samples.

### Statistical analysis

Chi-Square (*χ*^2^) test was used for all binary and categorical variables to assess differences between baseline characteristics by Self-reported trouble sleeping. We used *t* test for quantitative variable to assess differences between all examination indicators by self-reported trouble sleeping. Vitamin C is categorized into quartiles (“ ≤ 0.554”, “0.555 ~ 0.894”, “0.895 ~ 1.190”, “ > 1.19”), and the lowest quartile is considered as the reference group. We created four binary logistic regression models to determine associations between vitamin C and self-reported trouble sleeping. Model 1 was unadjusted; Model 2 Adjusted for gender, age and race/ethnicity based on model 1; Model 3 additionally adjusted for diabetes, hypertension, high cholesterol level, weak/failing kidneys, feeling depressed and little interest in doing things. based on Model 2; Model 4 additionally adjusted for weight, height, body mass index, upper arm length, arm circumference, waist circumference, total cholesterol, high-density lipoprotein cholesterol, ferritin, hypersensitive C-reactive protein, glycohemoglobin, transferrin receptor, alpha-carotene, cis-beta-carotene, gamma-tocopherol, total Lycopene, retinol and alpha-tocopherol based on Model 3. In addition, the association between vitamin C and self-reported trouble sleeping was further investigated by subgroup analysis stratified by gender, age, diabetes, hypertension and high cholesterol level. Finally, we used restricted cubic spline (RCS) model to detect the possible nonlinear dose–response relationship between vitamin C and self-reported trouble sleeping. The significant level is *P* < 0.05. R version 4.1.0 was used to conduct all statistical analysis.

## Results

### Baseline data of the study population

A total of 3227 participants were included in this study, of whom 49.1% were male and 50.9% were female, 23.5% were over 65 years of age. Moreover, among all participants, 29.1% had trouble sleeping and 70.9% hadn’t trouble sleeping. The baseline characteristics of all participants by self-reported trouble sleeping were showed in Tables 1 and 2, from which we can find statistically significant differences in gender, age, race/ethnicity, diabetes, hypertension, high cholesterol level, weak/failing kidneys, feeling depressed, little interest in doing things, weight, body mass index, upper leg length, upper arm length, arm circumference, waist circumference, hypersensitive C-reactive protein, glycohemoglobin, vitamin C, alpha-carotene, cis-beta-carotene, gamma-tocopherol, total Lycopene, retinol and alpha-tocopherol (all *P* < 0.05).

**Table 1:** Baseline characteristics of the NHANES 2017–2018 study sample[*N*(%)].

	*N*	No trouble sleeping	Trouble sleeping	*χ*^2^ value	*P* value
Overall	3227	2289 (70.9)	938 (29.1)		
Gender					
Male	1585	1178 (51.5)	407 (43.4)	17.352	< 0.001
Female	1642	1111 (48.5)	531 (56.6)		
Age					
≤ 65 Years	2469	1794 (78.4)	675 (72.0)	15.227	< 0.001
> 65 Years	758	495 (21.6)	263 (28.0)		
Race/ethnicity					
Mexican American	418	327 (14.3)	91 (9.7)	62.799	< 0.001
Other Hispanic	296	214 (9.3)	82 (8.7)		
Non-Hispanic White	1191	753 (32.9)	438 (46.7)		
Non-Hispanic Black	748	544 (23.8)	204 (21.7)		
Other Race	574	451 (19.7)	123 (13.1)		
Little interest in doing things					
Not at all	2433	1850 (80.8)	583 (62.2)	134.576	< 0.001
Several days	521	305 (13.3)	216 (23.0)		
More than half the days	161	84(3.7)	77(8.2)		
Nearly every day	112	50 (2.2)	62 (6.6)		
Feeling depressed					
Not at all	2468	1890 (82.6)	578 (61.6)	175.616	< 0.001
Several days	528	298 (13.0)	230 (24.5)		
More than half the days	133	61 (2.7)	72 (7.7)		
Nearly every day	98	40(1.7)	58 (6.2)		
Diabetes					
No	2718	1990 (86.9)	728 (77.6)	43.554	< 0.001
Yes	509	299 (13.1)	210 (22.4)		
Hypertension					
No	2037	1609 (70.3)	428 (45.6)	173.871	< 0.001
Yes	1190	680 (29.7)	510 (54.4)		
High cholesterol level					
No	2071	1579 (69.0)	492 (52.5)	79.079	< 0.001
Yes	1156	710 (31.0)	446 (47.5)		
Weak/failing kidneys					
No	3113	2236 (97.7)	877 (93.5)	32.240	< 0.001
Yes	114	53 (2.3)	61 (6.5)		
Vitamin C					
Q1 (≤ 0.554 mg/dL)	807	517 (22.6)	290 (30.9)	24.910	< 0.001
Q2(0.555 mg/dL ~ 0.894 mg/dL)	809	593 (25.9)	216 (23.0)		
Q3 (0.895 mg/dL ~ 1.190 mg/dL)	805	594 (26.0)	211 (22.5)		
Q4 (> 1.190 mg/dL)	806	585 (25.5)	221 (23.6)

**Table 2:** Demographic and clinical indicators of participants[mean ± SD].

Variables	Overall	No trouble sleeping	Trouble sleeping	*t* value	*P* value
Age	51.05 ± 17.29	49.59 ± 17.58	54.70 ± 16.01	− 7.995	< 0.001
Weight	82.67 ± 21.79	80.92 ± 20.59	86.95 ± 23.94	− 6.752	< 0.001
Height	166.73 ± 10.09	166.84 ± 10.12	166.48 ± 10.01	0.923	0.356
Body mass index	29.62 ± 6.88	28.94 ± 6.38	31.27 ± 7.73	− 8.132	< 0.001
Upper leg length	39.10 ± 3.85	39.29 ± 3.85	38.63 ± 3.79	4.401	< 0.001
Upper arm length	37.40 ± 2.85	37.32 ± 2.86	37.59 ± 2.83	− 2.454	0.014
Arm Circumference	33.41 ± 5.14	33.04 ± 4.92	34.31 ± 5.53	− 6.129	< 0.001
Waist Circumference	100.58 ± 16.67	98.77 ± 15.91	104.99 ± 17.64	− 9.349	< 0.001
Total cholesterol	4.90 ± 1.07	4.89 ± 1.05	4.91 ± 1.13	− 0.570	0.568
HDL-C	1.38 ± 0.40	1.38 ± 0.39	1.37 ± 0.42	1.051	0.293
Ferritin	159.17 ± 195.85	155.38 ± 170.36	168.41 ± 247.12	− 1.717	0.086
HS-CRP	3.83 ± 6.94	3.52 ± 6.30	4.58 ± 8.24	− 3.515	< 0.001
Glycohemoglobin	5.82 ± 1.03	5.78 ± 0.99	5.93 ± 1.12	− 3.445	< 0.001
Vitamin C	0.90 ± 0.49	0.92 ± 0.47	0.86 ± 0.53	2.800	0.005
Transferrin receptor	39.69 ± 23.10	39.84 ± 25.10	39.34 ± 17.27	0.561	0.575
Alpha-carotene	5.42 ± 9.27	5.76 ± 10.10	4.59 ± 6.73	3.843	< 0.001
Cis-beta-carotene	1.19 ± 1.39	1.24 ± 1.45	1.07 ± 1.23	3.293	0.001
Gamma-tocopherol	182.86 ± 99.29	178.02 ± 92.38	194.69 ± 113.59	− 3.986	< 0.001
Total lycopene	38.96 ± 19.53	39.49 ± 19.69	37.65 ± 19.08	2.421	0.016
Retinol	52.93 ± 16.27	52.08 ± 15.41	55.03 ± 18.04	− 4.397	< 0.001
Alpha-tocopherol	1255.48 ± 461.92	1238.60 ± 440.63	1296.68 ± 508.05	− 3.061	0.002

*HS-CRP*: Hypersensitive C-reactive protein, *HDL-C* high-density lipoprotein cholesterol.

### Association between vitamin C and trouble sleeping

After performing a binary logistic regression analysis, we found vitamin C in serum was a protective factor for trouble sleeping (Table 3). This relationship was significant in model 1(*OR* = 0.787; 95% *CI*:0.670 ~ 0.924), model 2(*OR* = 0.667; 95% *CI*:0.566 ~ 0.787) and model 3(*OR* = 0.809; 95% *CI*:0.681 ~ 0.960). After adjusting for all potential confounders(model 4), the negative association between vitamin C and trouble sleeping was still significant(*OR* = 0.816; 95% *CI*:0.669 ~ 0.995), suggesting that for every unit increase in vitamin C, the risk of trouble sleeping decreased by 18.4%. When using the lowest quartile of vitamin C as a reference group, individuals in the second to the fourth quartile had a lower risk of trouble sleeping after adjustment for all confounders. The ORs for trouble sleeping across increasing quartiles were 0.706(95% *CI*:0.558 ~ 0.894), 0.738(95% *CI*:0.577 ~ 0.942) and 0.707(95% *CI*:0.541 ~ 0.923) in the full-adjusted model(*P* for Trend < 0.05).

**Table 3:** Associations between vitamin C and trouble sleeping.

	Model 1 *OR*(95%*CI*)	Model 2 *OR*(95%*CI*)	Model 3 *OR*(95%*CI*)	Model 4 *OR*(95%*CI*)
vitamin C (Continuous variable)	0.787 (0.670 ~ 0.924)*	0.667 (0.566 ~ 0.787)*	0.809 (0.681 ~ 0.960)*	0.816 (0.669 ~ 0.995)*
vitamin C (classified variable )				
Q1(≤ 0.554 mg/dL)	Reference	Reference	Reference	Reference
Q2(0.555 mg/dL ~ 0.894 mg/dL)	0.649 (0.525 ~ 0.803)*	0.649 (0.523 ~ 0.804)*	0.677 (0.539 ~ 0.850)*	0.706 (0.558 ~ 0.894)*
Q3(0.895 mg/dL ~ 1.190 mg/dL)	0.633 (0.512 ~ 0.784)*	0.607 (0.489 ~ 0.753)*	0.720 (0.573 ~ 0.905)*	0.738 (0.577 ~ 0.942)*
Q4(> 1.190 mg/dL)	0.673 (0.545 ~ 0.832)*	0.553(0.444 ~ 0.689)*	0.696 (0.551 ~ 0.879)*	0.707 (0.541 ~ 0.923)*
*P* for Trend	< 0.001	< 0.001	0.003	0.012

Model 1. unadjusted; Model 2. Adjusted for gender, age and race/ethnicity; Model 3. Additionally adjusted for diabetes, hypertension, high cholesterol level, weak/failing kidneys, feeling depressed and little interest in doing things based on Model 2; Model 4. Additionally adjusted for weight, height, body mass index, upper leg length, upper arm length, arm circumference, waist circumference, total cholesterol, high-density lipoprotein cholesterol, ferritin, hypersensitive C-reactive protein, glycohemoglobin, transferrin receptor, alpha-carotene, cis-beta-carotene, gamma-tocopherol, total Lycopene, retinol and alpha-tocopherol based on Model 3.

*.*P* < 0.05.

### The non‑linear relationship between vitamin C and trouble sleeping

**Figure 2:**
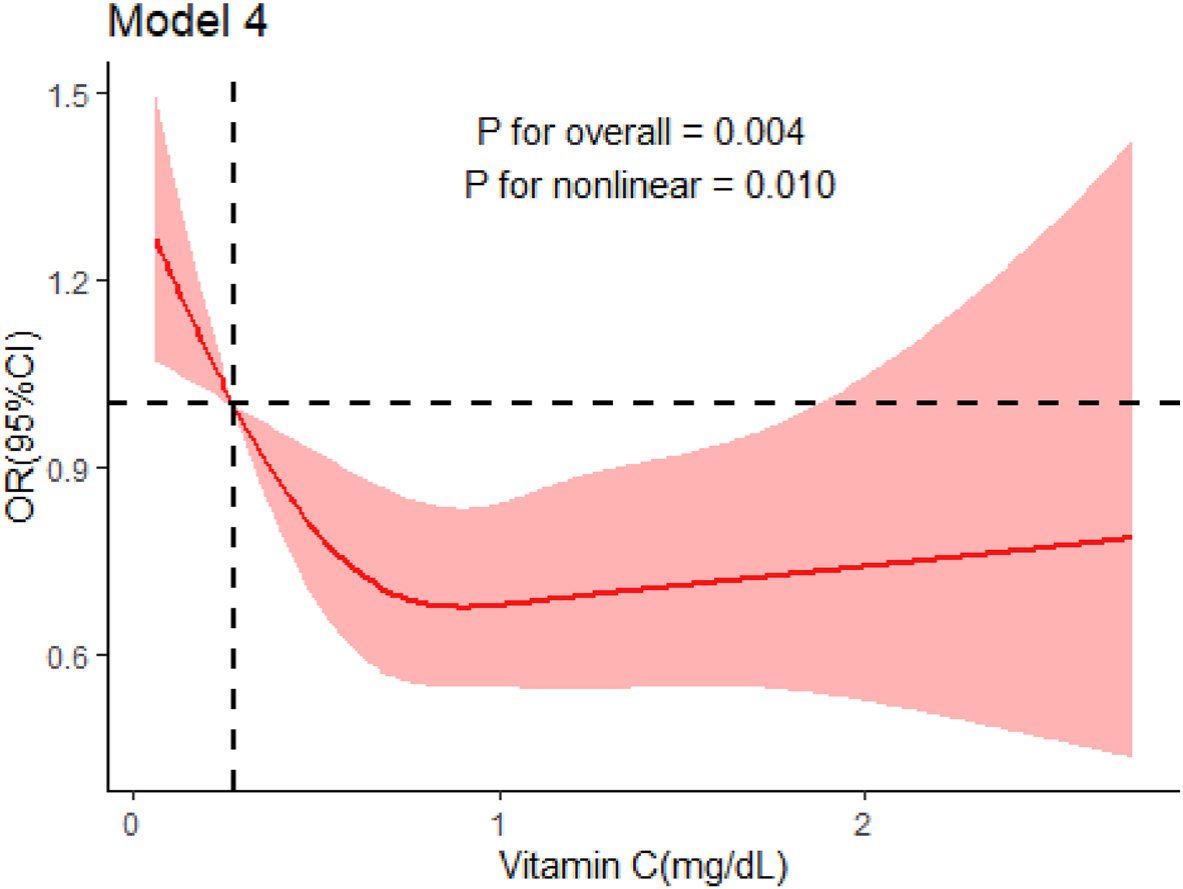
Restricted cubic spline of association between vitamin C and trouble sleeping after adjusting all confounders.

We used RCS model with four knots to simulate the non‑linear relationship between vitamin C in serum and trouble sleeping among all participants. After adjustment for all confounders, the RCS model showed significant nonlinear dose–response relationship between vitamin C and trouble sleeping (*P* value of nonlinear = 0.010). Figure 2 showed the results of RCS model.

### Subgroup analysis

Subgroup analysis results showed association between vitamin C in serum and trouble sleeping was statistically significant in female in model 1(*OR* = 0.647; 95% *CI*:0.523 ~ 0.801), model 2(*OR* = 0.579; 95% *CI*:0.466 ~ 0.718), model 3(*OR* = 0.727; 95% *CI*:0.579 ~ 0.912) and model 4(*OR* = 0.713; 95% *CI*:0.546 ~ 0.931), indicating vitamin C can decrease the risk of trouble sleeping in female. Meanwhile, similar association was also found in the group of age ≤ 65 years in model 1(*OR* = 0.639; 95% *CI*:0.521 ~ 0.784), model 2(*OR* = 0.591; 95% *CI*:0.480 ~ 0.727), model 3(*OR* = 0.757; 95% *CI*:0.607 ~ 0.945) and model 4(*OR* = 0.773; 95% *CI*:(0.600 ~ 0.996). For male and age > 65 years, this relationship was not statistically significant. Moreover, after adjusting all potential confounders, we also found vitamin C in serum was a protective factor for trouble sleeping in participants with high cholesterol level(*OR* = 0.738; 95% *CI*:0.548 ~ 0.994). Subgroup analysis results were showed in Table 4.

**Table 4:** Subgroup analysis of association between vitamin C in serum and trouble sleeping: models with vitamin C as a continuous variable.

	Model 1 *OR(*95*%CI*)	Model 2 *OR*(95*%CI)*	Model 3 *OR(*95*%CI*)	Model 4 *OR*(95*%CI)*
Gender				
Male	0.887 (0.688 ~ 1.143)	0.825 (0.639 ~ 1.064)	0.932 (0.717 ~ 1.213)	0.952 (0.705 ~ 1.286)
Female	0.647 (0.523 ~ 0.801)*	0.579 (0.466 ~ 0.718)*	0.727 (0.579 ~ 0.912)*	0.713 (0.546 ~ 0.931)*
Age				
≤ 65 years	0.639 (0.521 ~ 0.784)*	0.591 (0.480 ~ 0.727)*	0.757 (0.607 ~ 0.945)*	0.773 (0.600 ~ 0.996)*
> 65 years	0.982 (0.755 ~ 1.277)	0.913 (0.697 ~ 1.196)	0.978 (0.744 ~ 1.285)	0.970 (0.699 ~ 1.346)
Race/ethnicity				
Mexican American	1.009 (0.563 ~ 1.809)	0.900 (0.499 ~ 1.625)	0.984 (0.527 ~ 1.837)	0.767 (0.3481.692)
Other Hispanic	0.404 (0.204 ~ 0.800)*	0.325 (0.158 ~ 0.667)*	0.405 (0.188 ~ 0.872)*	0.443 (0.185 ~ 1.065)
Non-Hispanic White	0.870 (0.705 ~ 1.072)	0.738 (0.592 ~ 0.921)*	0.851 (0.676 ~ 1.071)	0.825 (0.628 ~ 1.085)
Non-Hispanic Black	0.645 (0.438 ~ 0.949)*	0.647 (0.437 ~ 0.956)*	0.861 (0.568 ~ 1.305)	0.932 (0.578 ~ 1.504)
Other Race	0.684 (0.430 ~ 1.087)	0.569 (0.346 ~ 0.935)*	0.708 (0.427 ~ 1.175)	0.794 (0.446 ~ 1.413)
Diabetes				
No	0.841 (0.702 ~ 1.007)	0.702 (0.583 ~ 0.846)*	0.825 (0.679 ~ 1.002)	0.828 (0.660 ~ 1.037)
Yes	0.705 (0.496 ~ 1.002)	0.670 (0.463 ~ 0.969)*	0.765 (0.525 ~ 1.113)	0.800 (0.517 ~ 1.238)
Hypertension				
No	0.810 (0.641 ~ 1.022)	0.693 (0.546 ~ 0.879)*	0.748 (0.587 ~ 0.953)*	0.786 (0.597 ~ 1.034)
Yes	0.832 (0.665 ~ 1.043)	0.814 (0.642 ~ 1.031)	0.933 (0.730 ~ 1.192)	0.891 (0.664 ~ 1.196)
High cholesterol level				
No	0.809 (0.647 ~ 1.012)	0.695( 0.553 ~ 0.874)*	0.855 (0.674 ~ 1.084)	0.875 (0.669 ~ 1.146)
Yes	0.695 (0.549 ~ 0.879)*	0.639 (0.501 ~ 0.815)*	0.780 (0.607 ~ 1.001)	0.738 (0.548 ~ 0.994)*

Model 1. unadjusted; Model 2. Adjusted for gender, age and race/ethnicity; Model 3. Additionally adjusted for diabetes, hypertension, high cholesterol level, weak/failing kidneys, feeling depressed and little interest in doing things based on Model 2; Model 4. Additionally adjusted for weight, height, body mass index, upper leg length, upper arm length, arm circumference, waist circumference, total cholesterol, high-density lipoprotein cholesterol, ferritin, hypersensitive C-reactive protein, glycohemoglobin, transferrin receptor, alpha-carotene, cis-beta-carotene, gamma-tocopherol, total Lycopene, retinol and alpha-tocopherol based on Model 3.

*.*P* < 0.05.

## Discussion

In this study, we found a link between vitamin C in serum and trouble sleeping, even after adjusting for gender, age, race/ethnicity, weight, height, total cholesterol, HDL-C, ferritin, HS-CRP and etc. Besides, the RCS model also showed that the risk of trouble sleeping decreased nonlinearly with increasing vitamin C in serum.

Vitamin C has multiple benefits for several diseases. In a review, vitamin C was reported to increase the death of cancerous cells by inhibiting BCL-2(B-cell lymphoma-2) expression and increasing the expression of BAX (BCL2-Association X) and caspase-3^18^. A cross-sectional study involving 5145 participants showed an inverse association between vitamin C intake and periodontitis^19^. A meta-analysis encompassing 25 observational studies and 91,966 participants demonstrated that dietary vitamin C intake was negatively associated with depression^20^. In addition, a large cohor study with a follow-up period of 20 years proved increasing vitamin C intake can decrease mortality rate from stroke^21^. However, studies of association between vitamin C in serum and trouble sleeping are lacking.

Our findings are in line with a previous studies reporting protective effects of vitamin C on trouble sleeping^22^. As an antioxidant, vitamin C played an essential role in neutralizing free radicals and reducing the risk of oxidative stress^23,24^. Oxidative stress can interfere with the normal function of the nervous system and affect sleep quality^25^. Therefore, the antioxidant properties of vitamin C may contribute to improving sleep quality. Recently, a cross-sectional study about the relationship between sleep duration and fruit/vegetable intakes showed reference sleepers(7–8 h/days) consumed more fruit and vegetable compared to short sleepers(< 7 h/days)^26^. A representative study including 4552 participants also demonstrated that the risk of non-restorative sleep decreased with increasing vitamin C intake (OR = 0.92; 95% CI = 0.86 ~ 0.99) ^27^. Furthermore, in a rat experimental model of obstructive sleep apnea (OSA), rats treated with vitamin C showed a lower concentration of advanced products of protein oxidation than non-treated rats^28^. The study results indicated vitamin C may be considered as a therapy for patients with OSA.

Our subgroup analysis shows that vitamin C in serum is a protective factor for trouble sleeping in female, but this relationship is not observed in males. Related studies to explain the sex differences are rare. For females, moderately increasing the intake of foods rich in vitamin C such as star fruit, guava, kiwi and broccoli may help improve sleep quality^9^. More interventions for vitamin C supplementation should be given to females. We also find that young participants (age ≤ 65 years) are likely to report trouble sleeping with decreased vitamin C levels in serum, but the similar link is not significant in participants aged > 65 years. There are no related studies to explain the reason for this phenomenon. The reason for the age differences may be due to differences in physiological and biochemical characterization between young and older adults. More studies should be conducted to explore the cause of age differences in the future. Moreover, In race/ethnicity stratified analysis, we found all subgroups had no significant association. These associations did not reach statistical significance because of reduced statistical power. More evidence should be explored in a larger sample population in future studies. Finally, our study also showed a negative association between vitamin C in serum and trouble sleeping in participants with high cholesterol level. This finding further fills the gap in the role of nutrients in sleep quality in groups with chronic diseases.

Our findings have clinically important implications. Trouble sleeping are a problem that bothers many people worldwide. Diet therapy is an efficient and convenient way for patients. Therefore, doctors can treat patients with trouble sleeping by vitamin C supplementation in the future.

There are several limitations in our study. NHANES was a cross-sectional study, which makes it difficult for us to know the causal relationship between vitamin C in serum and trouble sleeping. Therefore, our findings needed to be further confirmed by interventional or prospective studies. In the meantime, NHANES did not repeatedly measure vitamin C in serum, which might not reflect the long-term condition of participants. Another limiting aspect was more than half participants were excluded in our study due to incomplete information, which might cause bias.

## Conclusion

Our study suggests that vitamin C in serum is a protective factor for trouble sleeping, highlighting the importance of nutrition on the sleep quality. More studies are needed to clarify the link between vitamin C in serum and trouble sleeping in the future.

## Data Availability

The datasets used and/or analysed during the current study are available from the corresponding author on reasonable request.
